# Diterpenoid Alkaloids from the Chinese Traditional Herbal “Fuzi” and Their Cytotoxic Activity

**DOI:** 10.3390/molecules17055187

**Published:** 2012-05-04

**Authors:** Feng Gao, Yuan-Yuan Li, Dan Wang, Xing Huang, Qian Liu

**Affiliations:** Department of Chinese Traditional Herbal, Agronomy College, Sichuan Agricultural University, No.221, Huming Road, Wenjiang Region, Chengdu 611130, China

**Keywords:** Fuzi, *Aconitum carmichaeli* Debx., diterpenoid alkaloid, cytotoxicity

## Abstract

Ten diterpenoid alkaloids, including eight aconitine-type C_19_-diterpenoid alkaloids and two hetisine-type C_20_-diterpenoid alkaloids, were isolated from the secondary roots of *Aconitum carmichaeli* Debx., known as “Fuzi” in Chinese traditional herbal medicine. Their structures were established on the basis of their spectroscopic data and comparison with those of the literature. Among these alkaloids, chasmanine, oxonitine and 15-acetylsongoramine were isolated for the first time from this medicinal plant. The cytotoxic activity of the alkaloids were tested against several cell lines by the MTT method in which aconitine, hypaconitine, mesaconitne and oxonitine were found to strongly inhibit the growth of the HePG2 cell line, which showed that the existence and quantity of the ester groups have a significant influence on the cytotoxicity of the diterpenoid alkaloids.

## 1. Introduction

The secondary roots of *Aconitum carmichaeli* Debx. (Ranunculaceae), a famous Chinese traditional herbal known as “Fuzi”, found mainly distributed in Jiangyou City of Sichuan Province in China, have long been used as an analgesic, anti-inflammatory and anti-tumor agent [1]. Diterpenoid alkaloids classed as belonging to the C_18_-, C_19_- and C_20_-subtypes have been reported to be the main bioactive constituents of plants from the *Aconitum* genus [2]. Recently, the anti-tumor activity of these diterpenoid alkaloids with complex structures have attracted increasing interest [3,4].

Forty-seven alkaloids, including forty-three C_19_-diterpenoid alkaloids and four C_20_-diterpenoid alkaloids have been isolated from the *Aconitum carmichaeli* Debx [1,5]. In order to find more anti-tumor active substances, the secondary roots of *Aconitum carmichaeli* were phytochemically and pharmacologically investigated to obtain eight aconitine-type C_19_-diterpenoid alkaloids: aconitine (**1**), chasmanine (**2**), crassicauline A (**3**), oxonitine (**4**), deoxyaconitine (**5**), hypaconitine (**6**), mesaconitine (**7**), senbusine A (**8**), and two atisine-type C_20_-diterpenoid alkaloids, songoramine (**9**), 15-cetylsongoramine (**10**). The structures of isolated alkaloids were confirmed by the spectral analysis utilizing UV, IR, MS, and NMR, and comparison with the published data. Among them chasmanine (**2**), oxonitine (**4**) and 15-acetylsongoramine (**10**) were isolated for the first time from this medicinal plant. The cytotoxicity of isolated compounds was tested against several human cancer cell lines. Herein, we report the extraction, isolation, structural elucidation and cytotoxicity of diterpenoid alkaloids from the well-known Chinese traditional herbal medicine “Fuzi”.

## 2. Results and Discussion

**Figure 1:**
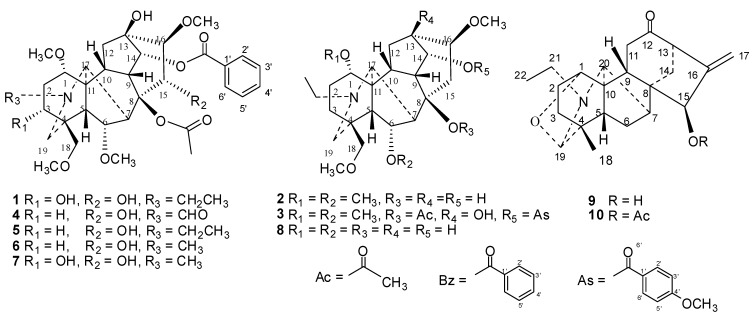
Structures of compounds **1**–**10**.

The ten isolated compounds were identified as aconitine (**1**), chasmanine (**2**), crassicauline A (**3**), oxonitine (**4**), deoxyaconitine (**5**), hypaconitine (**6**), mesaconitine (**7**), senbusine A (**8**), and two atisine-type C_20_-diterpenoid alkaloids, songoramine (**9**) and 15-cetylsongoramine (**10**), by comparison of their ^1^H- and ^13^C-NMR spectroscopic data with those reported in the literature. Figure 1 shows the structures of the alkaloids.

The cytotoxicity of the isolated ten alkaloids was evaluated against the human cancer cell lines HCT8, MCF7 and HePG2 and the results are shown in Table 1. Among these ten alkaloids, deoxyaconitine (**5**), oxonitine (**4**) and aconitine (**1**) exhibited the strongest cytotoxic activity against the HCT8, MCF7 and HePG2 cancer cell lines, respectively. Meanwhile, compounds **2**, **8**, **9** and **10** showed much lower activity than the other compounds.

**Table 1:** Cytotoxic data of compounds **1**–**10** from “Fuzi”.

Compds	IC_50_ (×10^−8^ M)			Compds	IC_50_ (×10^−8^ M)		
	HCT8	MCF7	HePG2		HCT8	MCF7	HePG2
**Taxol**	4.49 ± 0.83	0.71 ± 0.25	0.12 ± 0.47	**Taxol**	4.49 ± 0.83	0.71 ± 0.25	0.12 ± 0.47
**1**	8.12 ± 0.23	2.45 ± 1.02	0.85 ± 0.06	**6**	12.05 ± 0.76	6.46 ± 1.26	0.92 ± 0.06
**2**	>1,000	>1,000	>1,000	**7**	13.16 ± 1.58	4.57 ± 0.29	1.45 ± 0.01
**3**	16.45 ± 1.55	12.86 ± 3.52	2.36 ± 0.42	**8**	>1,000	>1,000	>1,000
**4**	29.48 ± 3.12	3.13 ± 0.14	8.61 ± 1.31	**9**	>1,000	>1,000	>1,000
**5**	5.14 ± 0.68	10.35 ± 1.24	9.21 ± 2.33	**10**	>1,000	>1,000	58.55 ± 9.17

HCT8: human colon cancer cell line; MCF7: human breast cancer cell line; HePG2: human liver cancer cell line.

Distinguishing differences in the anti-cancer bioactivity of the isolated compounds has been found in the MTT method. Analyzing the structures of the alkaloids and their anticancer data, we could conclude that: (1) the cytotoxic activities of C_19_-dierpenoid alkaloids were universally stronger than those of C_20_-dierpenoid alkaloids. For example, the only two C_20_-dierpenoid alkaloids **9** and **10** display extremely weak potency; (2) the number of ester groups of alkaloids has an extraordinarily important influence on their cytotoxicity. Obviously, the cytotoxicities of the C_19_-diterpenoid alkaloids containing two ester groups, such as aconitine (**1**), crassicauline A (**3**), oxonitine (**4**), deoxyaconitine (**5**), hypaconitine (**6**) and mesaconitine (**7**), were markedly stronger than those of chasmanine (**2**) and senbusine A (**8**) without the ester group substituent. With an additional acetyl group at C-15, 15-acetylsongoramine (**10**) also was found to be more potent than songoramine (**9**) against HePG2 cells. However, more pharmacological and biological experiments should be done to illustrate the interesting mechanism of action of how the ester groups influence the cytotoxic activity of diterpenoid alkaloids.

## 3. Experimental

### 3.1. General

^1^H- and ^13^C-NMR spectra were taken on a Varian Unity INOVA 400/45 MHz NMR spectrometer in CDCl_3_ with TMS as the internal standard. Silica gel H (Qingdao Sea Chemical Factory, Qingdao, China) was used as adsorbent for column chromatography. Spots on TLC (silica gel G, petroleum ether/acetone 1:1) were detected with modified Dragendorff’s reagent. Polyvinyl sulfuric ion-exchange resin (H-form, Chemical Factory of Nankai University, Tianjin, China) was used in the extraction of the crude alkaloids.

### 3.2. Plant Material Collection and Identification

The secondary roots of *Aconitum carmichaeli* were collected from Jiangyou City of Sichuan Province, China in September 2011, and indentified as “Fuzi” from the 2010 Chinese Pharmacopeia. A voucher specimen (No. 2011-9-16) was prepared from the entire plants of *Aconitum carmichaeli* and deposited in the herbarium of the Department of Chinese Traditional Herb, Agronomy College, Sichuan Agricultural University, China.

### 3.3. Extraction and Isolation

The powdered roots (1.8 kg) of *Aconitum carmichaeli* were percolated with 0.05 mol/L HCl (15 L). The aqueous acidic solution was basified with 10% aqueous NH_3_·H_2_O to pH 9–10 and then extracted with AcOEt (3 × 5 L). Removal of the solvent under reduced pressure afforded a total of 22.0 g of crude alkaloids as a yellowish amorphous powder. The total alkaloids (20 g) was chromatographed over SiO_2_-column, eluting with a petroleum ether/acetone (10:1 → 1:2) gradient system to give Fractions A-G. Fr.A (1.28 g) was further subjected to column chromatography (CC, silica gel, cyclohexane/acetone 8:1 → 4:1) to give songoramine (28 mg) and chasmanine (46 mg). Fr.B (4.85 g) was also further subjected to CC (silica gel, chloroform/acetone 95:5 → 80:20) to yield aconitine (460 mg), crassicauline A (92 mg) and 15-acetylsongoramine (31 mg). Further normal phased CC purification of Fr.C (5.42 g) was accomplished by elution with a cyclohexane/acetone (6:1 → 2:1) to afford hypaconitine (1.2 g), mesaconitine (840 mg), deoxyaconitine (49 mg) and senbusine A (18 mg). Fr.D (3.13 g) was chromatographed over SiO_2_-column with CHCl_3_/CH_3_OH (9:1 → 8:1) to provide oxonitine (45 mg).

### 3.4. NMR Spectroscopic Data

*Aconitine* (**1**) was obtained as white needle-like crystals. ^1^H-NMR (400 MHz, CDCl_3_): *δ*_H_ 1.14 (3H, t, *J* = 7.2 Hz, *N*-CH_2_CH_3_), 1.42 (3H, s, COCH_3_), 3.27, 3.37, 3.38, 3.42 (each 3H, s, 4 × OCH_3_), 5.02 (1H, d, *J* = 4.8 Hz, H-14), 7.55~8.12 (5H, m, H-Bz). ^13^C-NMR (100 MHz, CDCl_3_): *δ*_C_ 83.4 (C-1), 33.6 (C-2), 71.5 (C-3), 43.2 (C-4), 46.8 (C-5), 83.4 (C-6), 44.7 (C-7), 92.1 (C-8), 44.2 (C-9), 40.9 (C-10), 50.0 (C-11), 35.8 (C-12), 74.1 (C-13), 78.9 (C-14), 78.9 (C-15), 90.0 (C-16), 61.1 (C-17), 78.8 (C-18), 48.9 (C-19), 47.0 (*N*-CH_2_CH_3_), 13.3 (*N*-CH_2_CH_3_), 174.2 (8-CO-CH_3_), 21.4 (8-CO-CH_3_), 55.9 (1-OCH_3_), 59.1 (6-OCH_3_), 60.9 (16-OCH_3_), 57.9 (18-OCH_3_), 166.1 (Ar-CO), 129.8 (C-1'), 129.6 (C-2', C-6'), 128.6 (C-3', C-5'), 133.3 (C-4'). NMR data were in accordance with those reported in the literature [6], which confirmed that the isolated compound **1** was aconitine.

*Chasmanine* (**2**) was obtained as a white amorphous power. ^1^H-NMR (400 MHz, CDCl_3_): *δ*_H_ 1.07 (3H, t, *J* = 7.2 Hz, *N*-CH_2_CH_3_), 3.24, 3.31, 3.32, 3.34 (each 3H, s, 4 × OCH_3_), 4.15 (1H, t, *J* = 4.8 Hz, H-14*β*), 4.20 (1H, d, *J* = 6.8 Hz, H-6*β*). ^13^C-NMR (100 MHz, CDCl_3_): *δ*_C_ 85.9 (C-1), 25.8 (C-2), 35.0 (C-3), 39.2 (C-4), 48.5 (C-5), 82.2 (C-6), 52.4 (C-7), 72.4 (C-8), 50.0 (C-9), 45.3 (C-10), 50.1 (C-11), 28.4 (C-12), 37.5 (C-13), 75.3 (C-14), 39.0 (C-15), 82.0 (C-16), 62.3 (C-17), 80.6 (C-18), 53.7 (C-19), 49.1 (*N*-CH_2_CH_3_), 13.6 (*N*-CH_2_CH_3_), 56.2 (1-OCH_3_), 57.1 (6-OCH_3_), 55.9 (16-OCH_3_), 59.0 (18-OCH_3_). NMR data were in accordance with those reported in the literature [7], which confirmed that the isolated compound **2** was chasmanine.

*Crassicauline A* (**3**) was obtained as white lamellar crystals. ^1^H-NMR (400 MHz, CDCl_3_):*δ*_H_ 7.97 (2H, d, *J* = 8.8 Hz, H-2' and H-6'), 6.89 (2H, d, *J* = 8.8Hz, H-3' and H-5'), 4.85 (1H, d, *J* = 5.0 Hz, H-14b), 3.94 (1H, d, *J* = 6.8 Hz, H-6*β*), 3.84 (3H, s, Ar-OCH_3_), 3.50, 3.25, 3.23, 3.12 (each 3H, s, 4 × OCH_3_), 1.30 (3H, s, 8-OCOCH_3_), 1.06 (3H, t, *J* = 7.2 Hz, H-22). ^13^C-NMR (100 MHz, CDCl_3_): *δ*_C_ 83.8 (C-1), 26.4 (C-2), 34.9 (C-3), 39.4 (C-4), 49.0 (C-5), 83.1 (C-6), 45.1 (C-7), 85.7 (C-8), 49.6 (C-9), 41.1 (C-10), 50.4 (C-11), 36.0 (C-12), 75.0 (C-13), 78.7 (C-14), 39.5 (C-15), 84.9 (C-16), 62.1 (C-17), 80.5 (C-18), 53.8 (C-19), 49.3 (*N*-CH_2_CH_3_), 13.6 (*N*-CH_2_CH_3_), 167.0(8-OCOCH3), 21.8 (8-OCOCH_3_), 166.3 (14-CO-Ar), 122.8 (C-1'), 131.9 (C-2', C-6'), 113.9 (C-3', C-5'), 163.6 (C-4'), 59.2, 58.9, 57.9, 56.2, (4 × OCH_3_), 55.6 (As-OCH_3_). NMR data were in accordance with those reported in the literature [8], which confirmed that the isolated compound **3** was crassicauline A.

*Oxonitine* (**4**) was afforded as a white amorphous powder. ^1^H-NMR (400 MHz, CDCl_3_):*δ*_C_ 1.33 (3H, s, 8-OAc), 3.13, 3.25, 3.32, 3.78 (each 3H, s, 4 × OCH_3_), 4.87 (1H, d, *J* = 5.2 Hz, H-14*β*), 7.45~8.03 (5H, m, Ar-H), 8.10 (1H, s, *N*-COH). ^13^C-NMR (100 MHz, CDCl_3_): *δ*_C_ 82.8 (C-1), 34.2 (C-2), 69.9 (C-3), 42.2 (C-4), 49.2 (C-5), 79.8 (C-6), 50.9 (C-7), 90.3 (C-8), 43.1 (C-9), 40.5 (C-10), 49.7 (C-11), 33.6 (C-12), 74.2 (C-13), 78.8 (C-14), 78.5 (C-15), 90.0 (C-16), 57.9 (C-17), 74.8 (C-18), 38.7 (C-19), 163.1 (C-21), 55.6 (1-OCH_3_), 57.6 (6-OCH_3_), 61.1 (16-OCH_3_), 59.3 (18-OCH_3_), 172.3 (15-CO-CH_3_), 21.4 (15-CO-CH_3_), 166.2 (Ar-CO), 129.9 (C-1'), 129.6 (C-2', C-6'), 128.6 (C-3', C-5'), 133.2 (C-4'). The ^1^H- and ^13^C-NMR spectral data were in accordance with those reported jn the literature [9], which confirmed that the isolated compound **4** was oxonitine.

*Deoxyaconitine* (**5**) was obtained as white needle-like crystals. ^1^H-NMR (400 MHz, CDCl_3_):* δ*_H_ 1.08 (3H, t, *J* = 7.1 Hz, *N*-CH_2_CH_3_), 3.69, 3. 30, 3.28, 3.26 (each 3H, s, 4 × OCH_3_), 8.02 (2H, d, *J* = 7.5 Hz, H-2', 6'), 7.56 (1H, t, *J* = 7.5 Hz, H-4'), 7.43 (2H, t, *J* = 7.5 Hz, H-3', 5'). ^13^C-NMR (100 MHz, CDCl_3_): *δ*_C_ 85.3 (C-1), 26.4 (C-2), 35.1 (C-3), 39.1 (C-4), 48.9 (C-5), 83.5 (C-6), 49.3 (C-7), 82.1 (C-8), 47.0 (C-9), 42.1 (C-10), 48.8 (C-11), 37.1 (C-12), 74.9 (C-13), 78.5 (C-14), 80.5 (C-15), 91.1 (C-16), 61.7 (C-17), 80.0 (C-18), 53.6 (C-19), 50.4 (*N*-CH_2_CH_3_), 13.5 (*N*-CH_2_CH_3_), 56.3 (1-OCH_3_), 59.1 (6-OCH_3_), 61.5 (16-OCH_3_), 57.9 (18-OCH_3_), 166.3 (Ar-CO), 129.8 (C-1'), 129.8 (C-2', C-6'), 128.5 (C-3', C-5'), 133.2 (C-4'). NMR data were in accordance with those reported in the literature [10], which established that the isolated compound **5** was deoxyaconitine.

*Hypaconitine* (**6**) was afforded as white needle-like crystals. ^1^H-NMR (400 MHz, CDCl_3_): *δ*_H_ 1.35 (3H, s, 8-OAc), 2.31 (3H, s, NCH_3_), 3.70, 3.29, 3.25, 3.16 (each 3H, s, 4 × OCH_3_), 4.89 (1H, d, *J* = 4.8 Hz, H-14*β*), 7.40–8.03 (5H, m, H-Ar). ^13^C-NMR (100 MHz, CDCl_3_): *δ*_C_ 85.1 (C-1), 26.3 (C-2), 34.5 (C-3), 39.2 (C-4), 48.2 (C-5), 83.2 (C-6), 44.2 (C-7), 91.9 (C-8), 44.0 (C-9), 40.6 (C-10), 50.0 (C-11), 36.2 (C-12), 74.6 (C-13), 78.9 (C-14), 79.1 (C-15), 90.3 (C-16), 62.3 (C-17), 80.3 (C-18), 55.9 (C-19), 42.5 (C-21), 56.8 (1-OCH_3_), 58.1 (6-OCH_3_), 61.1 (16-OCH_3_), 59.0 (18-OCH_3_), 172.6 (8-COCH_3_), 21.4 (8-COCH_3_), 166.0 (14-CO-Ar), 129.5 (C-1'), 129.2 (C-2', C-6'), 128.3 (C-3', C-5'), 133.2 (C-4'). NMR data were in accordance with those reported by the literature [11], which proved that the isolated compound **6** was hypaconitine.

*Mesaconitine* (**7**) was isolated as a white amorphous powder. ^1^H-NMR (400 MHz, CDCl_3_):* δ*_H_ 1.36 (3H, s, 8-OAc), 2.33 (3H, s, NCH_3_), 3.74, 3.32, 3.29, 3.15 (each 3H, s, 4 × OCH_3_), 4.48 (1H, dd, *J*_1_ = 5.2 Hz, *J*_2_ = 2.8 Hz), 4.89 (1H, d, *J* = 5.2 Hz, H-14*β*), 7.41–8.04 (5H, m, H-Ar). ^13^C-NMR (100 MHz, CDCl_3_): *δ*_C_ 83.4 (C-1), 36.1 (C-2), 71.2 (C-3), 43.7 (C-4), 46.9 (C-5), 82.6 (C-6), 44.5 (C-7), 92.1 (C-8), 43.9 (C-9), 41.1 (C-10), 50.2 (C-11), 34.2 (C-12), 74.3 (C-13), 79.1 (C-14), 79.0 (C-15), 90.3 (C-16), 62.3 (C-17), 76.5 (C-18), 49.6 (C-19), 56.4, 58.0, 59.3, 61.2 (4 × OCH_3_), 42.6 (NCH_3_), 172.5 (8-COCH_3_), 21.6 (8-COCH_3_), 166.2 (14-CO-Ar), 129.9(C-1'), 129.8(C-2', C-6'), 128.7 (C-3', C-5'), 133.3 (C-4'). ^1^H- and ^13^C-NMR spectral data were in accordance with those reported in the literature [11], which confirmed that the obtained compound **7** was mesaconitine.

*Senbusine A* (**8**) was afforded as a white amorphous powder. ^1^H-NMR (400 MHz, CDCl_3_): *δ*_H_ 1.09 (3H, t, *J* = 7.2 Hz, *N*-CH_2_CH_3_), 3.28, 3.30, (each 3H, s, 2 × OCH_3_), 4.19 (1H, t, *J* = 5.2 Hz, H-14*β*). ^13^C-NMR (100 MHz, CDCl_3_): *δ*_C_ 72.1 (C-1), 29.3 (C-2), 29.6 (C-3), 37.9 (C-4), 48.3 (C-5), 72.7 (C-6), 55.3 (C-7), 76.4 (C-8), 45.6 (C-9), 40.4 (C-10), 49.8 (C-11), 29.7 (C-12), 44.1 (C-13), 75.6 (C-14), 42.3 (C-15), 82.1 (C-16), 63.6 (C-17), 80.3 (C-18), 57.0 (C-19), 48.2 (C-21), 13.0 (22), 56.3 (16-OCH_3_), 59.1 (18-OCH_3_). ^1^H- and ^13^C-NMR spectral data matched those reported by the literature [12], which demonstrated that the obtained compound **8** was senbsine A.

*Songoramine* (**9**) was isolated as a white amorphous powder. ^1^H-NMR (400 MHz, CDCl_3_): *δ*_H_ 0.85 (3H, s, H_3_-18), 1.04 (3H, t, *J* = 7.2 Hz, *N*-CH_2_CH_3_), 3.72 (1H, s, H-19*β*), 3.99 (1H, d, *J* = 7.2 Hz, H-1*β*), 4.41 (1H, m, H-15*α*), 5.21, 5.33 (each 1H, d, *J* = 1.6 Hz, H-17). ^13^C-NMR (100 MHz, CDCl_3_): *δ*_C_ 67.6 (C-1), 29.6 (C-2), 24.2 (C-3), 37.7 (C-4), 48.4 (C-5), 23.9 (C-6), 45.9 (C-7), 50.1 (C-8), 31.3 (C-9), 51.6 (C-10), 31.1 (C-11), 208.6 (C-12), 53.0 (C-13), 37.3 (C-14), 76.9 (C-15), 149.9 (C-16), 111.9 (C-17), 18.8 (C-18), 92.8 (C-19), 66.1 (C-20), 48.4 (C-21),14.4 (C-22). ^1^H- and ^13^C-NMR spectral data were in accordance with those reported in the literature [13], which vetified that the obtained compound **9** was songoramine.

*15-Acetylsongoramine* (**10**) was isolated as a white amorphous powder. ^1^H-NMR (400 MHz, CDCl_3_): *δ*_H_ 1.02 (3H, t, *J* = 7.2 Hz, *N*-CH_2_CH_3_), 0.84 (3H, s, 4-CH_3_), 2.14 (3H, s, 15-OAc), 4.95, 5.26 (each 1H, s, H_2_-17). ^13^C-NMR (100 MHz, CDCl_3_): *δ*_C_ 67.6 (C-1), 29.6 (C-2), 24.4 (C-3), 37.8 (C-4), 48.0 (C-5), 23.8 (C-6), 46.0 (C-7), 49.2 (C-8), 32.7 (C-9), 51.5 (C-10), 31.5 (C-11), 208.1 (C-12), 53.4 (C-13), 37.2 (C-14), 76.0 (C-15), 144.2 (C-16), 113.0 (C-17), 18.8 (C-18), 92.7 (C-19), 65.9 (C-20), 48.2 (C-21), 14.1 (C-22), 170.5 (15-CO-CH_3_), 21.4 (15-CO-CH_3_). NMR spectral data were agreeable with those reported in the literature [14], which confirmed that the obtained compound **10** was 15-acetylsongoramine.

### 3.5. Determination of Cell Viability by MTT Assay

Cells were plated in the RPMI 1640 with 10% fetal calf serum media on 96-well plates in a 100 μL total volume at a density of 1 × 10^4^ cells/mL. Triplicate wells were treated with media and tested compounds. The plates were incubated at 37 °C in 5% CO_2_ for 72 h. Cell viability was determined based on mitochondrial conversion of 3[4,5-dimethylthiazol-2-yl]-2,5-diphenyltetrazolium bromide (MTT, Sigma) to formazan. The amount of MTT converted to formazan is a sign of the number of viable cells. Each well was supplemented with 50 μL of a 1 mg/mL solution of MTT in incomplete media. The plates were incubated in 37 °C, 5% CO_2_ for another 4 h. The media was carefully removed from each well and 200 μL of DMSO was added. The plates were gently agitated until the color reaction was uniform and the OD_570_ was determined using a microplate reader (Wellscan MK3, Labsystems Dragon). Microsoft^®^ Excel 2000 was used for data analysis. Media-only treated cells served as the indicator of 100% cell viability. The 50% inhibitory concentration (IC_50_) was defined as the concentration that reduced the absorbance of the untreated wells by 50% of the control in the MTT assay.

## 4. Conclusions

Ten diterpenoid alkaloids were isolated from the poisonous traditional Chinese herbal drug prepared from the secondary roots of *Aconitum carmichaeli* Debx., known as “Fuzi”. Among them three alkaloids, including chasmanine, oxonitine and 15-acetylsongoramine, were isolated from this medicinal plant for the first time. The cytotoxic activities of these alkaloids were also tested against several human cancer cell lines, which showed that the ester groups play a considerably important role in the anticancer potency of those diterpenoid alkaloids.
